# Antibiotic Concentrations Decrease during Wastewater Treatment but Persist at Low Levels in Reclaimed Water

**DOI:** 10.3390/ijerph14060668

**Published:** 2017-06-21

**Authors:** Prachi Kulkarni, Nathan D. Olson, Greg A. Raspanti, Rachel E. Rosenberg Goldstein, Shawn G. Gibbs, Amir Sapkota, Amy R. Sapkota

**Affiliations:** 1 Maryland Institute for Applied Environmental Health, University of Maryland School of Public Health, 4200 Valley Drive, College Park, MD 20742, USA; prachik@umd.edu (P.K.); graspant@umd.edu (G.A.R.); rerosenb@umd.edu (R.E.R.G.); amirsap@umd.edu (A.S.); 2 University of Maryland Institute for Advanced Computer Studies, A.V. Williams Building, College Park, MD 20742, USA; nathandavidolson@gmail.com; 3 National Institute of Standards and Technology, Biosystems and Biomaterials Division, 100 Bureau Drive, Gaithersburg, MD 20899, USA; 4 School of Public Health-Bloomington, Indiana University Bloomington, 1025 E. 7th St., Bloomington, IN 47405, USA; gibbss@indiana.edu

**Keywords:** antibiotics, reclaimed water, wastewater treatment, liquid-chromatography-tandem mass spectrometry, public health

## Abstract

Reclaimed water has emerged as a potential irrigation solution to freshwater shortages. However, limited data exist on the persistence of antibiotics in reclaimed water used for irrigation. Therefore, we examined the fate of nine commonly-used antibiotics (ampicillin, azithromycin, ciprofloxacin, linezolid, oxacillin, oxolinic acid, penicillin G, pipemidic acid, and tetracycline) in differentially treated wastewater and reclaimed water from two U.S. regions. We collected 72 samples from two Mid-Atlantic and two Midwest treatment plants, as well as one Mid-Atlantic spray irrigation site. Antibiotic concentrations were measured using liquid-chromatography- tandem mass spectrometry. Data were analyzed using Mann-Whitney-Wilcoxon tests and Kruskal Wallis tests. Overall, antibiotic concentrations in effluent samples were lower than that of influent samples. Mid-Atlantic plants had similar influent but lower effluent antibiotic concentrations compared to Midwest plants. Azithromycin was detected at the highest concentrations (of all antibiotics) in influent and effluent samples from both regions. For most antibiotics, transport from the treatment plant to the irrigation site resulted in no changes in antibiotic concentrations, and UV treatment at the irrigation site had no effect on antibiotic concentrations in reclaimed water. Our findings show that low-level antibiotic concentrations persist in reclaimed water used for irrigation; however, the public health implications are unclear at this time.

## 1. Introduction

The use of reclaimed water (treated municipal wastewater) for landscape and agricultural irrigation is projected to rise in the United States (U.S.) [1]. However, research conducted on the safety of irrigating with reclaimed water has focused predominantly on the presence of microbial pathogens [1,2], heavy metals [1,2] and organics [1,2], with limited data available on the occurrence of pharmaceuticals (including antibiotics) in reclaimed water [3,4,5]. Antibiotics are extensively used in the U.S. for therapeutic use among humans, and therapeutic, prophylactic, and non-therapeutic use among food-production animals [6,7]. Consequently, most antibiotic residues enter wastewater due to incomplete metabolism or incorrect disposal [8]. Conventional wastewater treatment plants (WWTPs) in the U.S. are not designed to remove or monitor pharmaceuticals [9], resulting in the frequent detection of multiple antibiotics in municipal wastewater and treatment plant effluents [10,11]. 

Although the concentrations of antibiotics in wastewater effluent are relatively low [1], the combination of antibiotics, nutrients, and bacteria in reclaimed water (and in soil and plants subsequently irrigated with reclaimed water) could potentially result in the selection of antibiotic resistance among bacterial populations present in these environments [12,13]. Methicillin-resistant *Staphylococcus aureus* (MRSA) and vancomycin-resistant enterococci (VRE) have been detected in influent, activated sludge, secondary clarifier, post aeration, and effluent samples from U.S. WWTPs [14,15]. In addition, VRE have been detected at a U.S. reclaimed water spray irrigation site [16]. 

Antibiotics also have the potential to accumulate in soil and plants that have been irrigated with wastewater and reclaimed water [4,5,17,18]. Erythromycin was found to accumulate over five months in soil irrigated with reclaimed water [5], while six tetracyclines, 4-epianhydrotetracycline, doxycycline, and six quinolones [19] accumulated in soil during a one-month period of reclaimed water irrigation. However, there are few studies that have compared different wastewater treatment technologies with regard to their impacts on antibiotic concentrations in reclaimed water. In addition, to our knowledge there are little data regarding the impact of reclaimed water transport and additional reclamation site treatments on levels of antibiotics in reclaimed water.

Therefore, the goal of this study was to characterize antibiotic concentrations in differentially treated wastewater and reclaimed water from a spray irrigation site in order to evaluate the impact of treatment process variation and reuse site practices on the fate of antibiotic residues in reclaimed water intended for reuse. To our knowledge, this is the first study to analyze antibiotic concentrations throughout the treatment train (from wastewater influent to reclaimed water utilized at an associated reuse site for spray irrigation). Our findings inform the further exploration of treatment plant and reuse site practices, as well as future regulations, that may reduce the occurrence of antibiotics in reclaimed water.

## 2. Materials and Methods

### 2.1. Study Sites 

Wastewater samples collected from four U.S. wastewater treatment plants that supply treated effluent to reuse sites were included in this study: two WWTPs in the Mid-Atlantic region (previously described as Mid-Atlantic WWTP1 [14] and Mid-Atlantic WWTP2 [14]); and two WWTPs in the Midwest region (previously described as Midwest WWTP1 [14] and Midwest WWTP2 [14]). Reclaimed water samples from one spray irrigation site in the Mid-Atlantic region, previously described as Mid-Atlantic SI1 [16] (that receives treated effluent from Mid-Atlantic WWTP1 for landscape irrigation), were also tested in the study. All sites were chosen based on the willingness of the site operator to participate. A detailed description of each of the sites is included in Supplementary Information.

### 2.2. Sample Size and Description

Grab samples were collected throughout the treatment process (from May 2009 to October 2010), with sampling timing dependent on the availability of the WWTP operators and spray irrigation site managers. Schematics of our sampling locations have been previously described in Rosenberg et al. (2012) [14] and Carey et al. (2016) [16]. All samples were collected in 1L sterile polyethylene Nalgene^®^ Wide Mouth Environmental Sampling Bottles (Thermo Fisher Scientific, Waltham, MA, USA), transported to the laboratory at 4 °C, and stored at −80 °C until antibiotic residues could be isolated and quantified. A total of 72 samples were included in this analysis: 45 wastewater samples (16 from Mid-Atlantic WWTP1, 7 from Mid-Atlantic WWTP2, 11 from Midwest WWTP1, and 11 from Midwest WWTP2) and 27 reclaimed water samples from Mid-Atlantic SI1. In total, 15 influent, 4 activated sludge, 3 post-aeration, 6 secondary clarifier, 4 (lagoon) cell B and 13 effluent samples were collected from all WWTPs. From the Mid-Atlantic SI1 site, 6 samples were collected before UV treatment, 7 after UV treatment, 6 at the open-air storage pond inlet, and 8 at the pumphouse inlet. 

### 2.3. Extraction and Analysis of Antibiotic Concentrations

Nine antibiotics commonly used in the U.S. [20] and previously detected in wastewater samples [11] were analyzed: β lactams-ampicillin (AMP), oxacillin (OXA), and penicillin G (PEN); a macrolide–azithromycin (AZI); an oxazolidinone–linezolid (LIN); quinolones–ciprofloxacin (CIP), oxolinic acid (OXO), and pipemidic acid (PIP); and a tetracycline–tetracycline (TET). Antibiotic concentrations in all samples were quantified using a previously published method [21] (with modifications). A 10 μL aliquot of a methanol stock solution containing 10 μg/mL of surrogate standard (Linezolid-d3, Toronto Research Chemical Inc., Toronto, Canada, Cat # L466502) was added to a 200 mL aliquot of each sample, followed by thorough mixing and equilibration. All samples were then extracted using Oasis HLB (60 mg) cartridges (Waters Corp, Milford, MA, USA), conditioned with 3 mL methanol followed by a 3 mL water rinse. The samples were loaded under minimal vacuum using Visiprep 12-port Vacuum Manifolds (Sigma-Aldrich, St. Louis, MO, USA). Cartridges were then washed with 1 mL of water containing 5% methanol by volume and analytes were eluted with 6 mL of acetonitrile with 0.2% formic acid, followed by 3 mL of methanol:acetone mix (50:50; vol:vol) under minimal vacuum. Each extract was dried under nitrogen at 40 °C and reconstituted in 1 mL of acetonitrile:0.1% formic acid mix (50:50; vol:vol) followed by the addition of a 10 µL aliquot of 10 μg/mL internal standard (OxolinicAcid-d5, Toronto Research Chemical Inc., Toronto, Canada). High performance liquid chromatography tandem mass spectrometry (HPLC-MS/MS) was used to detect and quantify antibiotics using an Applied Biosystem ABI3000 tandem mass spectrometer with positive electrospray ionization, and chromatographic separation was achieved by an Xterra MS C18 2.5 µm, 2.1 × 50 mm column (Waters Corporation, Milford, MA, USA) with a pre-column filter (Phenomenex, Torrance, CA, USA). The list of antibiotics included in the analysis and their corresponding limits of detection (LOD) is provided in Supplementary Table S1.

### 2.4. Statistical Analysis

All statistical analyses were performed using R (version 3.2.4 2016 The R Foundation for Statistical Computing). Due to several samples with antibiotic concentrations below the LOD, certain antibiotics with very high concentrations (reflective of prescription patterns and, thus, considered representative of true sample concentrations), and small sample sizes at some WWTPs, a conservative, but robust, non-parametric rank-based approach was used for analysis [22]. Differences between groups were determined using the non-parametric Mann-Whitney-Wilcoxon test, or Kruskal Wallis test, based on the number of groups being compared [22]. The Bonferroni correction was used to adjust *p*-values when conducting multiple comparisons. In all cases, *p*-values ≤ 0.05 were defined as statistically significant, except when Bonferroni corrections were employed.

## 3. Results and Discussion

### 3.1. Antibiotic Concentrations in Influent Samples from All WWTPs

**Figure 1:**
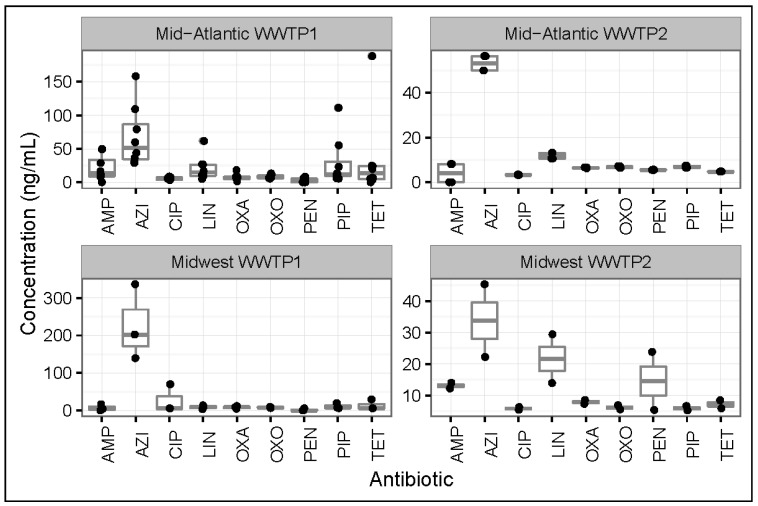
Concentrations (ng/mL) of antibiotics in influent samples collected from all four wastewater treatment plants (WWTPs) included in the study. AMP = Ampicillin; AZI = Azithromycin; CIP = Ciprofloxacin; LIN = Linezolid; OXA = Oxacillin; OXO = Oxolinic Acid; PEN = Penicillin; PIP = Pipemidic Acid; TET = Tetracycline.

Figure 1 summarizes the antibiotic concentrations detected in influent samples across all WWTPs. Antibiotic detection ranges in ng/mL were as follows: ampicillin (<LOD to 49.7), oxacillin (1.39 to 18), penicillin (<LOD to 23.8), azithromycin (22.2 to 336), ciprofloxacin (3.28 to 69.5), oxolinic acid (5.35 to 9.43), pipemidic acid (5.23 to 55.1), linezolid (3.05 to 61.5), and tetracycline (<LOD to 188).

Azithromycin was detected at the highest concentrations compared to all antibiotics in influent samples recovered from all WWTPs, with the highest concentration occurring in influent samples collected from Midwest WWTP1. Concentrations of azithromycin in both the Midwest WWTP1 and the Mid-Atlantic WWTP1 influents were, on average, an order of magnitude higher than those detected at the other WWTPs. Azithromycin concentrations were also the highest of all antibiotics analyzed (in influent, activated sludge, and effluent samples) at another U.S. wastewater treatment plant located in Kentucky [23]. Azithromycin, which is the most commonly prescribed human-use antibiotic in the U.S. [24,25] and has been found at fairly high concentrations in biosolids [26] with a relatively long half-life in biosolid-amended soil [26], may have entered Mid-Atlantic WWTP1 through domestic and hospital wastewater [14,15] and Midwest WWTP1 through domestic and agriculturally-influenced stormwater [14,15]. 

β-lactams were found at the lowest concentrations (compared to other antibiotics) in influent samples from all WWTPs, with 20% of influent samples containing ampicillin below the LOD and 33% of influent samples containing penicillin G below LOD. Despite being one of the most highly used classes of antibiotics in the U.S. [24], β-lactams are not usually found in high concentrations in influent samples [11] (due to chemical hydrolysis in the influent stream, or cleavage of the unstable β-lactam ring by β-lactamases [11]). 

### 3.2. Antibiotic Concentrations in Effluent Samples from All WWTPs

**Figure 2:**
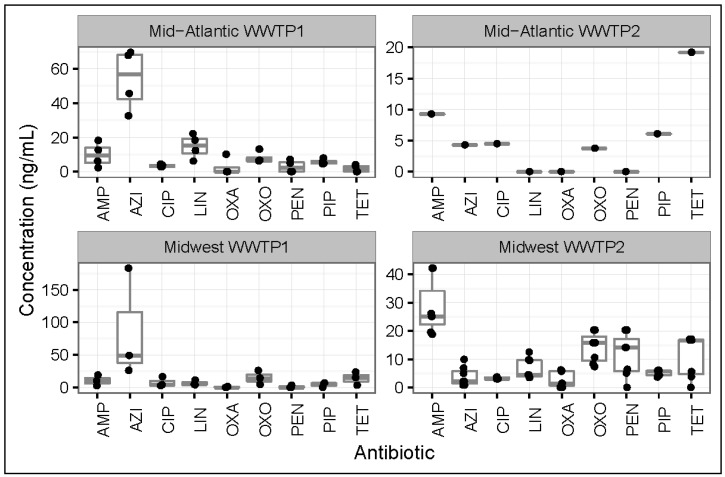
Concentrations (ng/mL) of antibiotics in effluent samples collected from all four wastewater treatment plants (WWTPs) included in the study. AMP = Ampicillin; AZI = Azithromycin; CIP = Ciprofloxacin; LIN = Linezolid; OXA = Oxacillin; OXO = Oxolinic Acid; PEN = Penicillin; PIP = Pipemidic Acid; TET = Tetracycline.

The antibiotic concentrations detected in effluent samples from all WWTPs are displayed in Figure 2. Antibiotic detection ranges in ng/mL were as follows: ampicillin (2.31 to 42.2), oxacillin (<LOD to 10.1), penicillin (<LOD to 20.3), azithromycin (0.82 to 183), ciprofloxacin (2.71 to 16.4), oxolinic acid (<LOD to 7.94), pipemidic acid (3.76 to 26), linezolid (<LOD to 22.1), and tetracycline (<LOD to 23.6). Oxacillin, penicillin G, tetracycline, and pipemidic acid occurred at concentrations below the LOD in 54%, 46%, 23%, and 8% of all effluent samples (from all WWTPs, respectively). The β-lactams would have undergone further cleavage and hydrolysis during wastewater treatment [11], while tetracycline (due to its extremely high sludge-wastewater partition coefficient [27]) may have been adsorbed into activated sludge. 

### 3.3. Differences in Antibiotic Concentrations between Same-Day Influent versus Effluent Samples 

**Figure 3:**
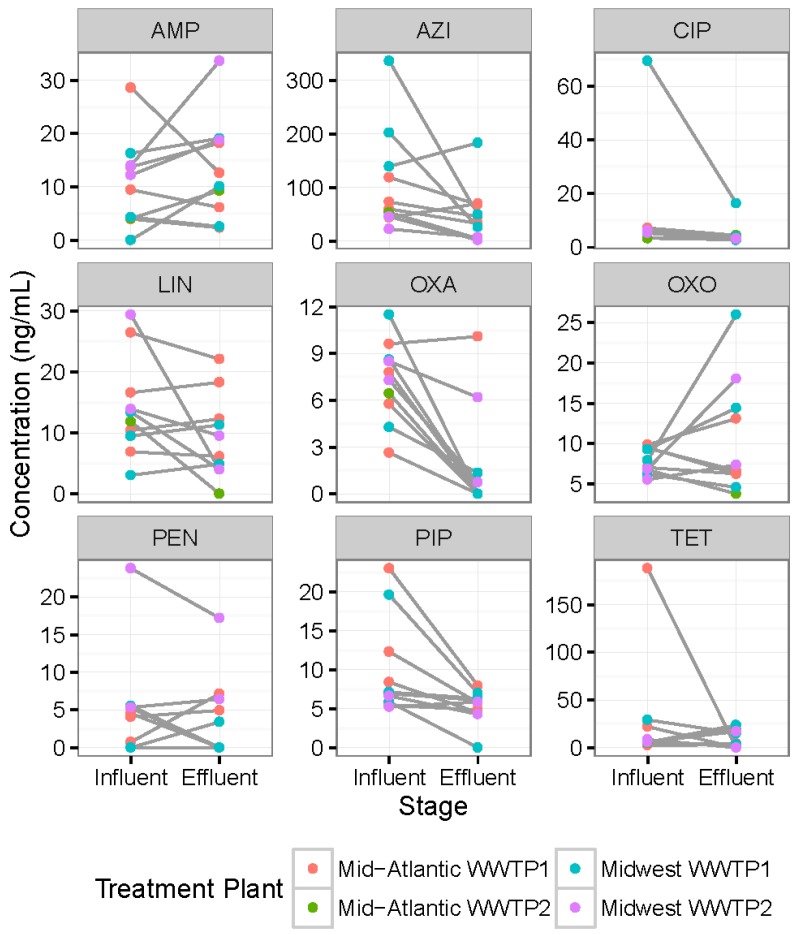
Differences in antibiotic concentrations (ng/mL) between influent versus effluent samples collected on the same day from each of the four wastewater treatment plants (WWTPs) included in the study. AMP = Ampicillin; AZI = Azithromycin; CIP = Ciprofloxacin; LIN = Linezolid; OXA = Oxacillin; OXO = Oxolinic Acid; PEN = Penicillin; PIP = Pipemidic Acid; TET = Tetracycline.

Antibiotic concentration differences between influent and effluent samples collected on the same day from each of the WWTPs are illustrated in Figure 3. In general, concentrations of most antibiotics were lower in the effluent samples compared to influent samples, with differences (at marginal significance) between influent and effluent concentrations observed only for oxacillin (W = 54, *p*-value = 0.004) and pipemidic acid (W = 53, *p*-value = 0.006). To account for multiple comparisons, *p*-values at or below 0.005 were considered to be statistically significant. Statistically significant differences for just two of the nine antibiotics analyzed may have been due to the cross sectional nature of the grab samples and our irregular access to some WWTPs (which was dictated by plant operators). 

### 3.4. Regional Differences between Antibiotic Concentrations in Influents and Effluents

Antibiotic concentration differences between Mid-Atlantic and Midwest WWTP influents can be seen in Supplementary Figure S1. Generally, most influent antibiotic concentrations were similar between the two regions (except for azithromycin concentrations (which were higher—though not statistically significantly) in the Midwest WWTP influents) compared to the Mid-Atlantic treatment plant influents. Azithromycin levels may have been higher in the raw influent of Midwest WWTPs [14,15] compared to Mid-Atlantic plants, because Midwest influents were comprised of both domestic wastewater and agriculturally-influenced stormwater. Since the Midwest plants are located in rural areas where biosolids are applied to agricultural land [14,15], runoff from this land during rain events could have increased levels of azithromycin in the waste stream. 

Antibiotic concentration differences between effluents from the Midwest and Mid-Atlantic regions are shown in Supplementary Figure S2. In spite of most antibiotics being at similar concentrations in all influent samples, ampicillin, oxacillin, oxolinic acid, penicillin G, and tetracycline were found at higher concentrations in the effluents from Midwest WWTPs, while azithromycin and linezolid were found at higher concentrations in the effluents from Mid-Atlantic WWTPs. None of these differences, however, were statistically significant.

The observed variability in antibiotic removal could be attributed to treatment process variations; namely, the treatment plant capacity, nature of influent, and type of tertiary treatment. Other differences could have been due to WWTP reactor type and solid-retention time (SRT), both of which impact microbial population characteristics of activated sludge [27,28]. Pharmaceutical degradation is achieved by nitrifying bacteria (through the production of monooxygenase (including ammonia monooxygenase) and dioxygenase enzymes [29]), which increase with longer SRT [30] and occur at higher concentrations in activated sludge from a nitrification reactor compared to a conventional activated sludge reactor [6]. Variability could have been due to the type of activated sludge reactor present at each plant [26,31]. Although all four plants in our study contained an activated sludge process, the types varied from a conventional continuous activated sludge reactor (Mid-Atlantic WWTP1), aeration tanks (Mid-Atlantic WWTP2) and a sequencing batch reactor (Midwest WWTP2) to activated sludge lagoons (Midwest WWTP2). SRT variability also could have influenced the observed differences between plants; however, this information was not obtained during the study. 

### 3.5. Differences in Antibiotic Concentrations across Wastewater Treatment Processes

**Figure 4:**
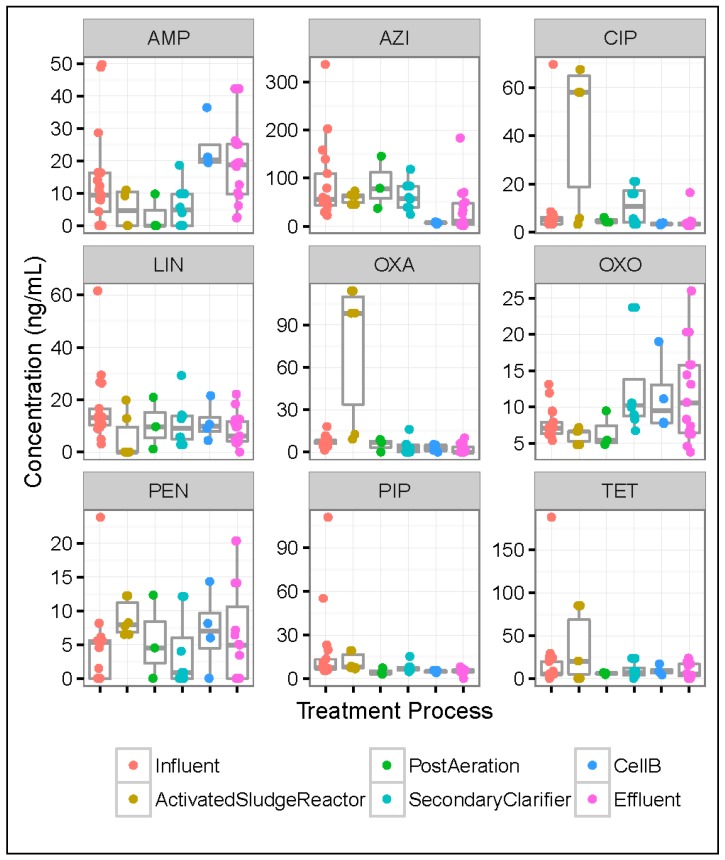
Differences in concentrations (ng/mL) of antibiotics across treatment processes used at all the wastewater treatment plants (WWTPs) included in the study. AMP = Ampicillin; AZI = Azithromycin; CIP = Ciprofloxacin; LIN = Linezolid; OXA = Oxacillin; OXO = Oxolinic Acid; PEN = Penicillin; PIP = Pipemidic Acid; TET = Tetracycline.

Antibiotic concentration differences across all treatment processes utilized at all WWTPs are described in Figure 4. In general, most antibiotics partitioned into samples from various treatment processes based on the chemical and physical properties of the class to which they belong. Statistically significant differences were found only for oxacillin (between influent and effluent samples (W = 28, *p*-value = 0.0002)) and activated sludge and effluent samples (W = 89, *p*-value = 0.0005). To account for multiple comparisons, *p*-values at or below 0.0005 were considered to be statistically significant. 

Ciprofloxacin and pipemidic acid were relatively abundant in activated sludge samples due to their non-volatility [27] and fairly high sludge-wastewater partition coefficient [27]. These antibiotics are also resistant to microbial degradation [28,32] but susceptible to photochemical degradation [28,32]. However, the large amounts of organic matter in activated sludge may have blocked light and resulted in reduced photochemical degradation.

Azithromycin (despite having a relatively low sludge-wastewater partition coefficient [11]) and oxacillin and penicillin G (despite being more prone to hydrolysis [11]) were also found at high concentrations in activated sludge. Azithromycin may have continued to persist in activated sludge due to its high influent concentrations. Activated sludge samples from another U.S. treatment plant in Kentucky also contained high azithromycin concentrations [23]. Higher than expected concentrations of other antibiotics (including β-lactams) may have also occurred due to interactions with proteins, nucleic acids, and polysaccharide cell-wall components of activated sludge bacteria [28], along with bonding and complexation with lipids, fats, and other particulate matter in activated sludge, allowing compounds with low octanol–water and sludge–wastewater coefficients to easily adsorb into activated sludge [28]. Tetracycline (a non-volatile compound [27] with a high sludge-wastewater partition coefficient [27] and the ability to undergo polarization or complexation with solid particles [28,33]) was found at unexpectedly low concentrations in activated sludge samples, possibly due to the relatively low therapeutic use of tetracycline among humans [11]. 

### 3.6. Differences in Antibiotic Concentrations from Mid-Atlantic WWTP1 to Mid-Atlantic SI1

**Figure 5:**
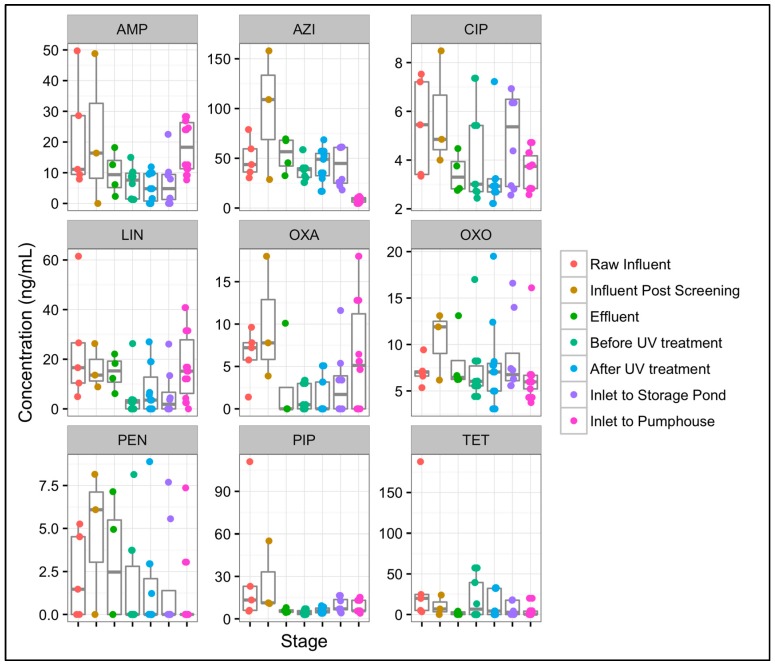
Changes in antibiotic concentrations (ng/mL) as wastewater travels from the influent at Mid-Atlantic wastewater treatment plant 1 (Mid-Atlantic WWTP1), undergoes tertiary treatment, and is then piped to Mid-Atlantic spray irrigation site 1 (Mid-Atlantic SI1) for reuse. The sequential order of flow is as follows: (1) Raw influent; (2) Influent post screening; (3) Effluent; (4) Before UV treatment; (5) After UV treatment; (6) Inlet to storage pond; and (7) Inlet to pumphouse. AMP = Ampicillin; AZI = Azithromycin; CIP = Ciprofloxacin; LIN = Linezolid; OXA = Oxacillin; OXO = Oxolinic Acid; PEN = Penicillin; PIP = Pipemidic Acid; TET = Tetracycline.

Figure 5 illustrates the changes in antibiotic concentrations in samples obtained sequentially from the influent at Mid-Atlantic WWTP1 through the Mid-Atlantic SI1 pumphouse sprinkler. For all antibiotics, transport from the WWTP (“Effluent”) to the spray irrigation site (“Before UV treatment”) resulted in virtually unchanged median concentrations. The only observed decrease in median concentration was for azithromycin (56.6 ng/mL in “Effluent” to 38.6 ng/mL in “Before UV treatment”). Similarly, the median concentrations of almost all of the antibiotics remained unchanged after UV treatment at the spray irrigation site. Open-air storage at the spray irrigation site resulted in a decrease in the median concentration of azithromycin (44.85 ng/mL to 8.79 ng/mL), but almost all other antibiotics remained at virtually unchanged levels before and after storage. 

Ampicillin concentrations, however, were statistically significantly higher in “Inlet to pumphouse” samples compared to “After UV treatment” samples (W = 14, *p*-value = 0.0006), indicating that storage in an open-air pond may have contributed to this increase. In addition, azithromycin concentrations were statistically significantly different between: “Inlet to storage pond” samples and “Inlet to pumphouse” samples (W = 112, *p*-value = 0.0001); “After UV treatment” samples and “Inlet to pumphouse” samples (W = 154, *p*-value < 0.0001); “Before UV treatment” samples and “Inlet to pumphouse” samples (W = 140, *p*-value < 0.0001); and Mid-Atlantic WWTP1 influent samples and “Inlet to pumphouse” samples (W = 112, *p*-value = 0.0001). These differences provided evidence of an overall trend of decreasing azithromycin concentrations as effluent flowed from WWTP1 and was subsequently stored at the spray irrigation site. To account for multiple comparisons, *p*-values at or below 0.0006 were considered statistically significant. 

Distribution system characteristics, such as residual chlorine, pH, temperature, biofilm community structure, and dissolved organic matter (parameters we were unable to assess) could have influenced antibiotic concentrations during transport; however, our data showed that the effects were negligible. On-site UV radiation treatment was performed at a wavelength (254 nm) that has previously been found to be ineffective at reducing antibiotic concentrations [27]. Azithromycin may have undergone photodegradation in the storage pond, influenced by direct photolysis (due to direct excitation from solar radiation) or indirect photolysis (due to interaction with reactive intermediates generated by humic acids [34]).

### 3.7. Limitations

The main limitations of this study were the convenience sample of WWTPs (where plants were chosen based on the willingness of each plant to participate), the collection of grab samples, and unequal sample sizes resulting from limited access to some collection sites. Furthermore, since we could only include one spray irrigation site in our study, our findings may not be applicable to all U.S. spray irrigation sites. However, by studying four conventional WWTPs across two regions, our observations could be representative of multiple types of conventional wastewater treatment processes commonly employed in different regions of the U.S. 

### 3.8. Public Health Impacts and Future Research

Antibiotics have the potential to exert selective pressure on existing bacterial communities within WWTPs [6] and in reclaimed water [13], potentially contributing to increased levels of antibiotic resistance within these environments [14]. Both MRSA and VRE have been detected in the same WWTP effluents that were tested in this study and sent to reuse applications [14,15], and VRE was detected in the reclaimed water that we tested from the Mid-Atlantic spray irrigation site [16]. Thus, it is possible that the trace levels of antibiotics that we observed in the wastewater and reclaimed water samples (in the range of <LOD to 336 ng/mL in influent samples, and <LOD to 183 ng/mL in effluent or reclaimed water samples) could have contributed to the selection of bacteria that are resistant to those specific antibiotics. In addition, the variable impact of different treatment technologies and storage conditions on antibiotic degradation is also a potential concern, particularly since some antibiotics (ciprofloxacin and ofloxacin) have been shown to be genotoxic [35]. Our data show that antibiotics remain at low levels in reclaimed water (<LOD to 183 ng/mL), but the effect of chronic human exposures to complex mixtures of antibiotics and other pharmaceuticals in reclaimed water is unclear and deserves further study [36]. 

## 4. Conclusions

We confirmed that conventional continuous activated sludge processes alone may not effectively remove antibiotics from municipal wastewater. We also observed the persistence of antibiotics in reclaimed water at a spray irrigation site, in spite of on-site UV treatment (with levels in the range of <LOD to 68.6 ng/mL depending on the antibiotic). If conventionally-treated municipal wastewater is increasingly used for downstream purposes such as irrigation, then additional, cost-effective, onsite technologies may need to be developed in order to reduce the occurrence of persisting contaminants (including antibiotics) in the reclaimed water and prevent the dissemination of these contaminants into the environment and human populations.

## Supplementary Materials

The following are available online at www.mdpi.com/1660-4601/14/6/668/s1, Figure S1: Differences in antibiotic concentrations (ng/mL) between influent samples collected from Mid-Atlantic versus Midwest wastewater treatment plants (WWTPs), Figure S2: Differences in antibiotic concentrations (ng/mL) between effluent samples collected from Mid-Atlantic versus Midwest wastewater treatment plants (WWTPs), Table S1: A list of the nine antibiotics analyzed with the corresponding mass-charge ratios (*m*/*z*) of their parent and daughter ions and limit of detection (LOD) values (ng/mL).

## Disclaimer

Certain commercial equipment, instruments, or materials are identified in this paper only to specify the experimental procedure adequately. Such identification is not intended to imply recommendation or endorsement by the NIST, nor is it intended to imply that the materials or equipment identified are necessarily the best available for the purpose. Official contribution of NIST; not subject to copyrights in USA.
