# Supplementary material for: Antibiotic Concentrations Decrease during Wastewater Treatment but Persist at Low Levels in Reclaimed Water
Source: Int J Environ Res Public Health. 2017 Jun 21;14(6):668. doi: 10.3390/ijerph14060668 (PMC5486354; doi:10.3390/ijerph14060668)
Supplement: Supplementary file 1 [file ijerph-14-00668-s001.pdf]

International Journal of
*Environmental Research and Public Health*

MDPI

**Supporting Text**

# Antibiotic Concentrations Decrease during Wastewater Treatment but Persist at Low Levels in Reclaimed Water

**Prachi Kulkarni, Nathan D. Olson, Greg A. Raspanti, Rachel E. Rosenberg Goldstein, Shawn G. Gibbs, Amir Sapkota, Amy R. Sapkota**

Number of Pages (Including Cover Page) – 3
Contents of Supporting Text
Supporting Text
References for Supporting Text

## Detailed description of all sampling sites included in the study

All sites were chosen based on the willingness of the site operator to participate. Mid-Atlantic WWTP1 is an urban tertiary wastewater treatment plant processing 681,390 $m^3$ of wastewater per day with a peak capacity of 1.51 x 10^6 $m^3/d$[1,2]. The influent includes domestic and hospital wastewater[1,2]. Treatment steps at this plant are screens, primary clarifier, activated sludge reactors, secondary clarifier, sand filters, chlorination (dose of 2 mg/L to 3 mg/L), de-chlorination (with sodium bisulfite) and effluent discharge (chlorine residual of <0.1 mg/L)[1,2]. Effluent from this plant is piped to a landscaping site (Mid-Atlantic SI1) for reuse in spray irrigation[2,3]. Mid-Atlantic SI1 performs on-site treatment and storage prior to spray irrigation[2,3]. On-site treatment includes screening (double-walled aluminum screen) and ultraviolet (UV) disinfection (minimum of 30,000 $\mu W/cm^2$ with 254 nm wavelength UV bulbs)[2,3]. The UV treated reclaimed water is then pumped to an open air storage pond (peak capacity 15,142 $m^3$) at a rate of 3.29 $m^3/d$[2,3]. Water from the storage pond is then pumped to spray heads based on irrigation needs[2,3]. Site employees use backpack sprayers to apply reclaimed water to locations not reached by spray heads[3].

Mid-Atlantic WWTP2 is a suburban tertiary treatment plant processing 7,570 $m^3$ of wastewater per day with a peak capacity of 45,425 $m^3/d$[1,2]. The influent includes domestic and hospital wastewater[1,2]. Treatment steps at this plant are screens, primary clarifier, primary aeration tank, secondary aeration tank, secondary clarifier, multimedia filter, chlorination (dose of 2 mg/L to 3 mg/L), de-chlorination (with sodium bisulfite) and effluent discharge (chlorine residual of <0.1 mg/L)[1,2]. Effluent from this plant is transported to a landscaping site for reuse via spray irrigation[1,2].

Midwest WWTP1 is a rural tertiary treatment plant processing 1,363 $m^3$ of wastewater per day with a peak capacity of 10,978 $m^3/d$[1,2]. The influent at this plant includes domestic wastewater and agriculturally influenced stormwater[1,2]. Treatment steps at this plant are screens, activated sludge lagoons, clarifiers, seasonal chlorination (in June, July and August; dose of 4 mg/L) and de-chlorination, and effluent discharge (chlorine residual of 0 mg/L)[1,2]. Effluent from this plant is transported to a landscaping site for reuse via spray irrigation[1,2].

Midwest WWTP2 is a rural tertiary treatment plant processing 1,439 $m^3$ of wastewater per day with a peak capacity of 7,571 $m^3/d$[1,2]. The influent includes domestic, food production and agriculturally influenced wastewater[1,2]. Treatment steps at this plant are screens, sequencing batch reactor, lagoon cell A, lagoon cell B, lagoon cell C, lagoon cell D, lagoon cell E and effluent discharge[1,2]. There is no on-site disinfection and unchlorinated effluent from this plant is transported to an agricultural site for irrigation of animal feed crops[1,2].

## Supporting Figures and Tables

Table S1: A list of the nine antibiotics analyzed with the corresponding mass-charge ratios (*m*/*z*) of their parent and daughter ions and limit of detection (LOD) values (ng/mL)

Figure S1: Differences in antibiotic concentrations (ng/mL) between influent samples collected from Mid-Atlantic versus Midwest wastewater treatment plants (WWTPs)

Figure S2: Differences in antibiotic concentrations (ng/mL) between effluent samples collected from Mid-Atlantic versus Midwest wastewater treatment plants (WWTPs)

**Table S1**: A list of the nine antibiotics analyzed with the corresponding mass-charge ratios (*m*/*z*) of their parent and daughter ions and their limit of detection (LOD) values (ng/mL)

| Antibiotic | Parent Ion (m/z)[a] | Daughter Ion (m/z)[a] | LOD (ng/mL) |
|---|---|---|---|
| Ampicillin (AMP) | 366.7 | 206.9 | 0.0242 |
| Azithromycin (AZI) | 375.0 | 113.1 | 0.0092 |
| Ciprofloxacin (CIP) | 331.5 | 287.4 | 0.0131 |
| Linezolid (LIN) | 337.5 | 295.4 | 0.0217 |
| Oxacillin (OXA) | 402.0 | 158.2 | 0.0201 |
| Oxolinic Acid (OXO) | 261.1 | 243.0 | 0.0213 |
| Penicillin G (PEN) | 334.6 | 158.2 | 0.0308 |
| Pipemidic Acid (PIP) | 303.4 | 215.9 | 0.0279 |
| Tetracycline (TET) | 445.0 | 409.9 | 0.0107 |

[a]mass-charge ratio

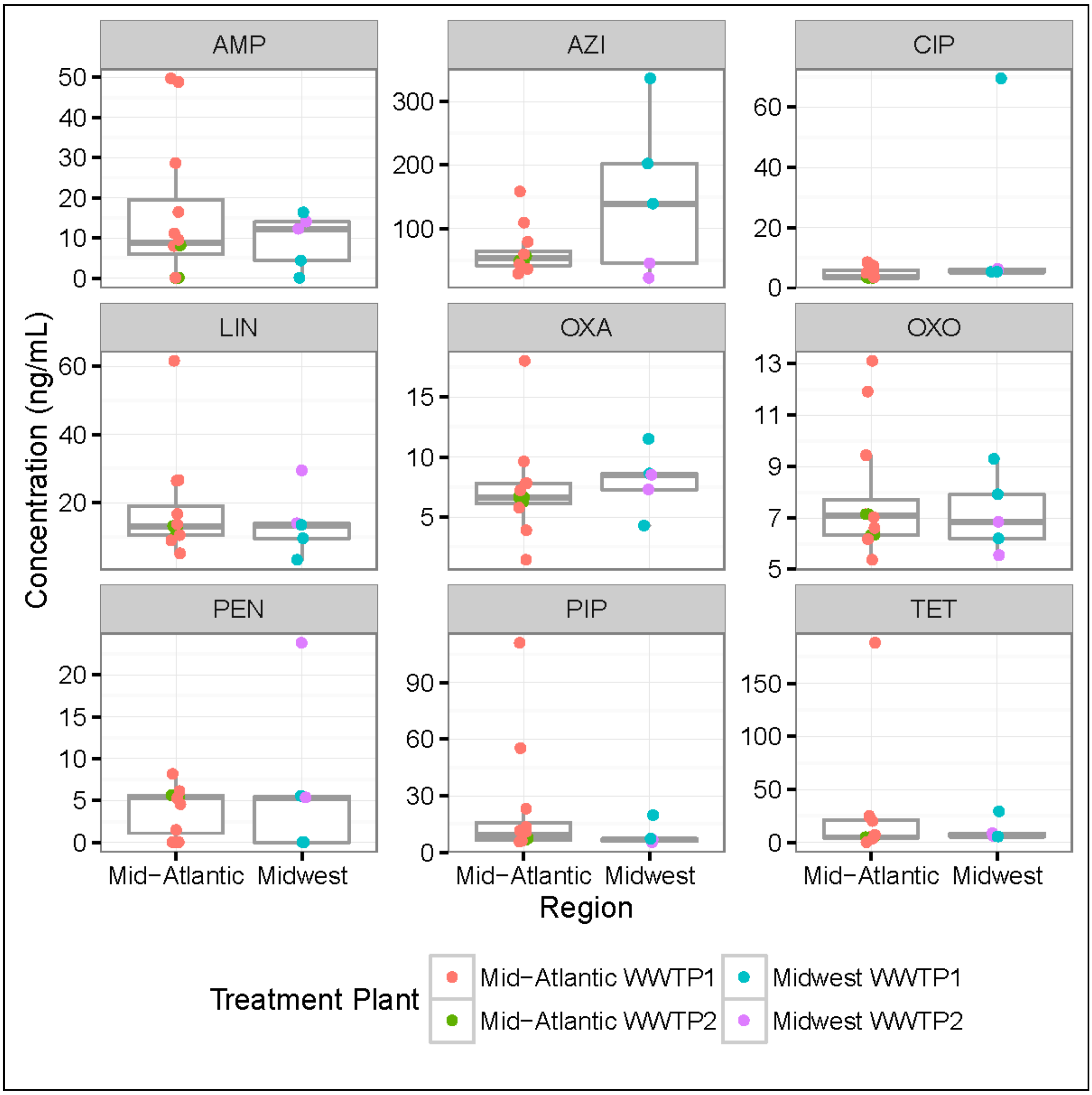


**Figure S1**: Differences in antibiotic concentrations (ng/mL) between influent samples collected from Mid-Atlantic versus Midwest wastewater treatment plants (WWTPs)

AMP = Ampicillin; AZI = Azithromycin; CIP = Ciprofloxacin; LIN = Linezolid;

OXA = Oxacillin; OXO = Oxolinic Acid; PEN = Penicillin; PIP = Pipemidic Acid;

TET = Tetracycline

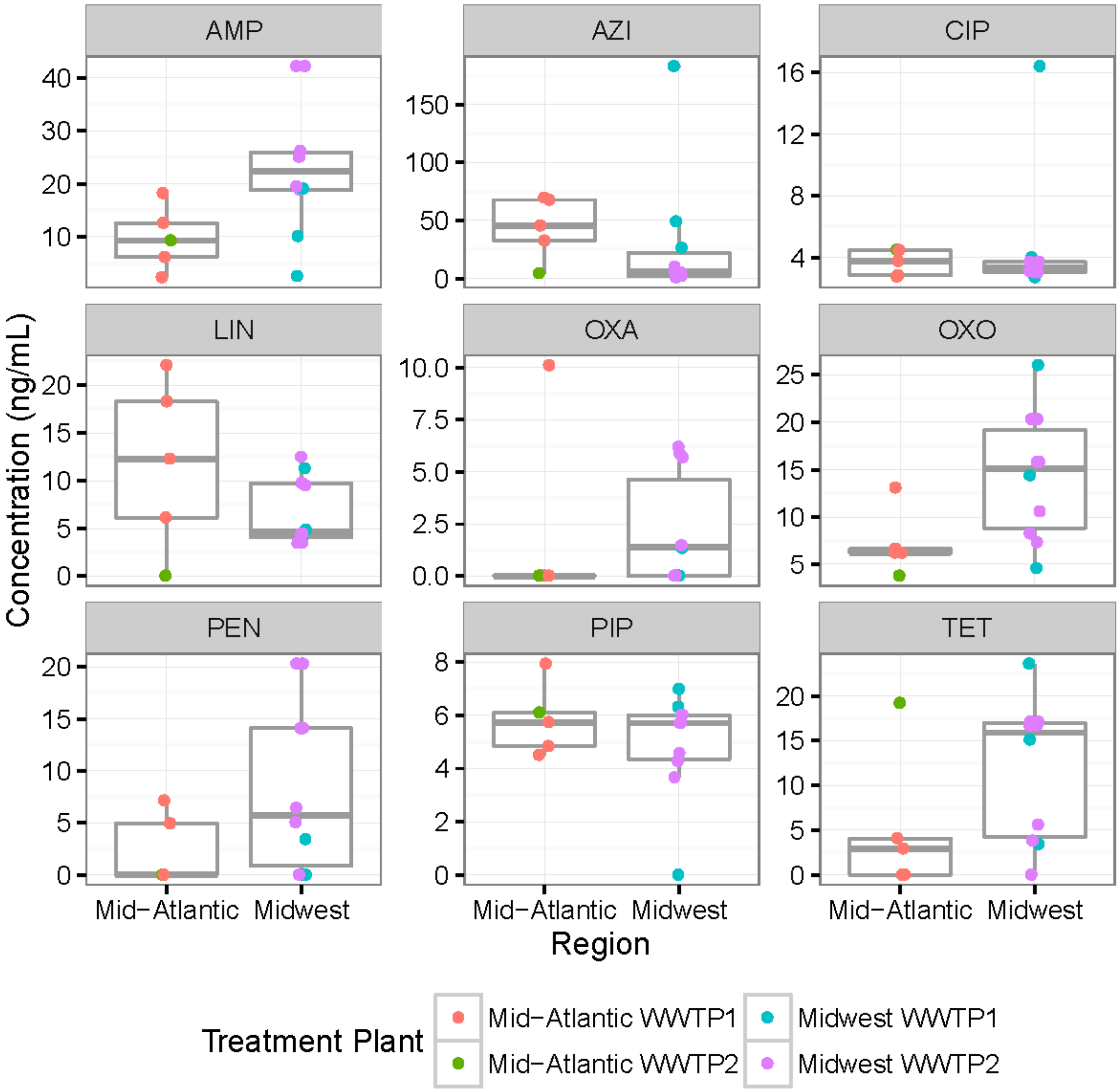


**Figure S2**: Differences in antibiotic concentrations (ng/mL) between effluent samples collected from Mid-Atlantic versus Midwest wastewater treatment plants (WWTPs)

AMP = Ampicillin; AZI = Azithromycin; CIP = Ciprofloxacin; LIN = Linezolid;

OXA = Oxacillin; OXO = Oxolinic Acid; PEN = Penicillin; PIP = Pipemidic Acid;

TET = Tetracycline

**References**

1. Rosenberg Goldstein RE, Micallef SA, Gibbs SG, et al. Methicillin-resistant Staphylococcus aureus (MRSA) detected at four U.S. wastewater treatment plants. *Environ Health Perspect*. 2012;120(11):1551-1558. doi:10.1289/ehp.1205436.
2. Rosenberg Goldstein RE, Micallef SA, Gibbs SG, et al. Detection of vancomycin-resistant enterococci (VRE) at four U.S. wastewater treatment plants that provide effluent for reuse. *Sci Total Environ*. 2014;466-467:404-411. doi:10.1016/j.scitotenv.2013.07.039.
3. Rosenberg Goldstein RE, Micallef SA, Gibbs SG, et al. Occupational exposure to Staphylococcus aureus and Enterococcus spp. among spray irrigation workers using reclaimed water. *Int J*

*Environ Res Public Health*. 2014;11(4):4340-4355. doi:10.3390/ijerph110404340.

© 2017 by the authors; licensee MDPI, Basel, Switzerland. This article is an open access article distributed under the terms and conditions of the Creative Commons Attribution (CC-BY) license (http://creativecommons.org/licenses/by/4.0/).
